# Automatic detection of main pancreatic duct dilation and pancreatic parenchymal atrophy based on a shape feature in abdominal contrast-enhanced CT images

**DOI:** 10.1117/1.JMI.12.1.014504

**Published:** 2025-01-31

**Authors:** Shintaro Ambo, Ryo Hirano, Chihiro Hattori

**Affiliations:** Canon Medical Systems Corporation, Otawara, Japan

**Keywords:** pancreas, main pancreatic duct, CT, deep learning, segmentation

## Abstract

**Purpose:**

The purpose of this study was to develop and evaluate an algorithm for calculating a shape feature to automatically detect both main pancreatic duct dilation (MPDD) and pancreatic parenchymal atrophy (PPA) in abdominal contrast-enhanced CT (CE-CT) images.

**Approach:**

The proposed algorithm for the automatic detection of MPDD and PPA is composed of five processes: coarse pancreas segmentation, fine pancreas segmentation, main pancreatic duct (MPD) segmentation, centerline estimation, and shape feature calculation. First, the pancreas region is segmented by a deep learning convolutional neural network (CNN). Then, the MPD region is segmented inside the pancreatic region by the deep learning CNN. Next, centerline estimation is performed using Dijkstra’s rooting algorithm. Finally, in shape feature calculation, the cross-sectional area ratio of the pancreatic duct to the pancreatic parenchyma (DP ratio) is calculated in all cross sections perpendicular to the identified centerline, and the 90th percentile value of the DP ratio for all cross sections (90th DP ratio) is calculated. The detection performance of the 90th DP ratio for MPDD and PPA was evaluated using 56 abdominal CE-CT images available as public data.

**Results:**

The average of the 90th DP ratio was 0.059 in 48 cases with MPDD and 0.007 in eight cases without MPDD (p<0.001) and 0.074 in 31 cases with PPA and 0.023 in 25 cases without PPA (p<0.001).

**Conclusions:**

We have developed an algorithm for calculating an automatically measurable shape feature called the 90th DP ratio for the detection of MPDD and PPA.

## 1 Introduction

Most pancreatic cancers are diagnosed at an advanced stage, and they are among the most lethal malignant neoplasms.^1^^,^^2^ It is therefore important to develop effective screening methods for the detection of early-stage pancreatic cancer. Various studies have been conducted using AI in the diagnosis of pancreatic cancer.^3^ In this study, we focused on the main pancreatic duct dilation (MPDD) and pancreatic parenchymal atrophy (PPA). MPDD and PPA are recognized as imaging findings of early pancreatic cancer.^4^^,^^5^ Various imaging findings, including MPDD and PPA, may be found in abdominal and chest contrast-enhanced computed tomography (CT) images acquired for unrelated reasons within 1 year prior to the diagnosis of pancreatic cancer, even though the imaging findings may not have been reported at the time the images were acquired.^6^ Miura et al. found no significant difference in the presence or absence of imaging findings related to the main pancreatic duct (MPD) between a group of patients with early-stage pancreatic cancer and a benign group but did find significant differences in the presence or absence of regional parenchymal atrophy and fat displacement.^7^ These results indicate that identifying PPA may be more useful than identifying MPDD for the early diagnosis of pancreatic cancer. Therefore, the detection of not only MPDD but also PPA is required for the diagnosis of early-stage pancreatic cancer. Abi Nader et al. proposed a deep learning method to detect MPDD automatically in portal venous CT images.^8^ Shen et al. proposed a segmentation model for dilated pancreatic ducts which is based on anatomical attention.^9^ Yamada et al. investigated the possibility of using contrast-enhanced CT (CE-CT) images for predicting PPA after steroid therapy.^10^ However, to our knowledge, few studies have reported on automatic detection of PPA and the automatic detection of both MPDD and PPA using a single indicator has also not yet been reported.

The purpose of this study was to develop and evaluate an algorithm for calculating a shape feature to automatically detect both MPDD and PPA in abdominal CE-CT images.

## 2 Materials and Methods

### 2.1 Datasets

In this study, a dataset was used to develop the algorithm for calculating an automatically measurable shape feature to detect both MPDD and PPA in abdominal CE-CT images and another dataset was used to evaluate the algorithm.

The dataset for developing the algorithm, which was composed of five private datasets and eight public datasets with a total of 2243 CT images which contain CE-CT and non-contrast CT, was used for the segmentation technique employed in our proposed algorithm (Table 1). Labels of the pancreatic region were created on the images used for coarse pancreatic segmentation and fine pancreatic segmentation, and labels on the MPD region were created on the images used for MPD segmentation. With regard to the private datasets, the CT images in the liver dataset were acquired at one Chinese hospital with the approval of the institutional review board and the CT images in the NCCC-PCDS, NCCC-Cyst, NCCC-MPDD, and NCCC-normal datasets were acquired at the National Cancer Center Hospital, Japan. Liver included abdominal CT images of four patients scanned for suspected liver disease, NCCC-PCDS included abdominal CE-CT images of 170 patients diagnosed with pancreatic cancer, NCCC-Cyst included abdominal CE-CT images of 29 patients diagnosed with pancreatic cysts, NCCC-MPDD included abdominal CE-CT images of 84 patients diagnosed with MPDD, and NCCC-normal included abdominal CE-CT images of five patients who did not have any pancreatic lesions. Ground truth regions of the development dataset were created by one of the following methods: using public ground truth datasets, manual annotating by engineers, manual annotating by a radiologist, reviewing by a radiologist to revise the manual annotation results by engineers, and reviewing by a radiologist or engineers to revise pseudo-labels generated by nnU-net.^18^

**Table 1:** Dataset for development.

Process	Dataset	Private/public	License	Image size (voxel)	Pixel size (mm)	Slice thickness (mm]	Annotation method	Training	Validation
Coarse pancreas segmentation	TotalSegmentator^11^	Public	CC-BY 4.0	47–499 × 89–430	1.5	1.5	Pseudo labels	900 cases	0 case
	C4KC-KiTS^12^	Public	CC-BY 3.0	512 × 512	0.44–0.98	0.5–5	Pseudo labels	104 cases	0 case
	MSD03^13^	Public	CC-BY-SA 4.0	512 × 512	0.56–1	0.70–5	Pseudo labels (90%), Radiologist (10%)	120 cases	0 case
	MSD08^13^	Public	CC-BY-SA 4.0	512 × 512	0.59–0.98	0.8–8	Pseudo labels	171 cases	0 case
	Liver	Private		512 × 512	0.78–0.89	0.8	Pseudo labels	4 cases	0 case
	TCIA-panNET^14^^,^^15^	Public	CC-BY 4.0	512 × 512	0.57–0.88	0.5–3	Engineer and radiologist	59 cases	0 case
	TCGA-LIHC^16^	Public	CC-BY 3.0	512 × 512	0.59–0.98	0.63–8.5	Pseudo labels	49 cases	0 case
Fine pancreas segmentation	Pancreas-CT^17^	Public	CC-BY 3.0	512 × 512	0.66–0.98	0.5–1	Public	66 cases	16 cases
	MSD07^13^	Public	CC-BY-SA 4.0	512 × 512	0.61–0.98	1.25–5.0	Public	225 cases	56 cases
	NCCC-PCDS	Private		512–1024 × 512–1024	0.29–0.74	1	Engineer	136 cases	34cases
Main pancreatic duct segmentation	MSD07^13^	Public	CC-BY-SA 4.0	512 × 512	0.61–0.98	1.25–5.0	Public	126 cases	34 cases
	NCCC-PCDS	Private		512–1024 × 512–1024	0.31–0.74	1	Engineer	22 cases	3 cases
	NCCC-Cyst	Private		1024 × 1024	0.31–0.39	1	Engineer	22 cases	7 cases
	NCCC-Normal	Private		1024 × 1024	0.31–0.43	1	Engineer	4 cases	1 case
	NCCC-MPDD	Private		512–1024 × 512–1024	0.31–0.68	1	Engineer	67 cases	17 cases

*Notes*: Meanings in the table of annotation methods; “Public” means using public Ground truth datasets. “Engineer” means annotated by engineers. “Radiologist” means annotated by a radiologist. “Engineer and radiologist” means reviewing by a radiologist to revise the manual annotation results by engineers. “Pseudo labels” means reviewing by a radiologist or engineers to revise pseudo labels generated by nnU-net.

The dataset for evaluating the algorithm, which was composed of two public datasets with a total of 56 abdominal CE-CT images, included annotations regarding the presence or absence of MPDD and PPA added by a radiologist with 4 years of post-residency experience (Table 2).^19^ The evaluation dataset included 31 abdominal CE-CT images with both MPDD and PPA, 17 with only MPDD and eight without both MPDD and PPA (Table 3). There were not any CT images with only PPA in the evaluation dataset.

**Table 2:** Dataset for evaluation.

Dataset	Private/public	License	Image size (voxel)	Slice thickness (mm)	Number
CPTAC-PDA^19^^,^^20^	Public	CC-BY 3.0	512 × 512	1 to 5	42 cases
MSD07^13^^,^^19^	Public	CC-BY-SA 4.0	512 × 512	2.5 to 5	14 cases

**Table 3:** Number of cases with imaging findings in the evaluation dataset.

Imaging findings		Number of cases
MPDD	PPA	
(+)	(+)	31
(+)	(−)	17
(−)	(+)	0
(−)	(−)	8

MPDD, main pancreatic duct dilation. PPA, pancreatic parenchymal atrophy. (+), presence. (−), absence.

### 2.2 Automatic Detection of Main Pancreatic Duct Dilation and Pancreatic Parenchymal Atrophy

#### 2.2.1 Concept of the proposed algorithm

**Fig. 1:**
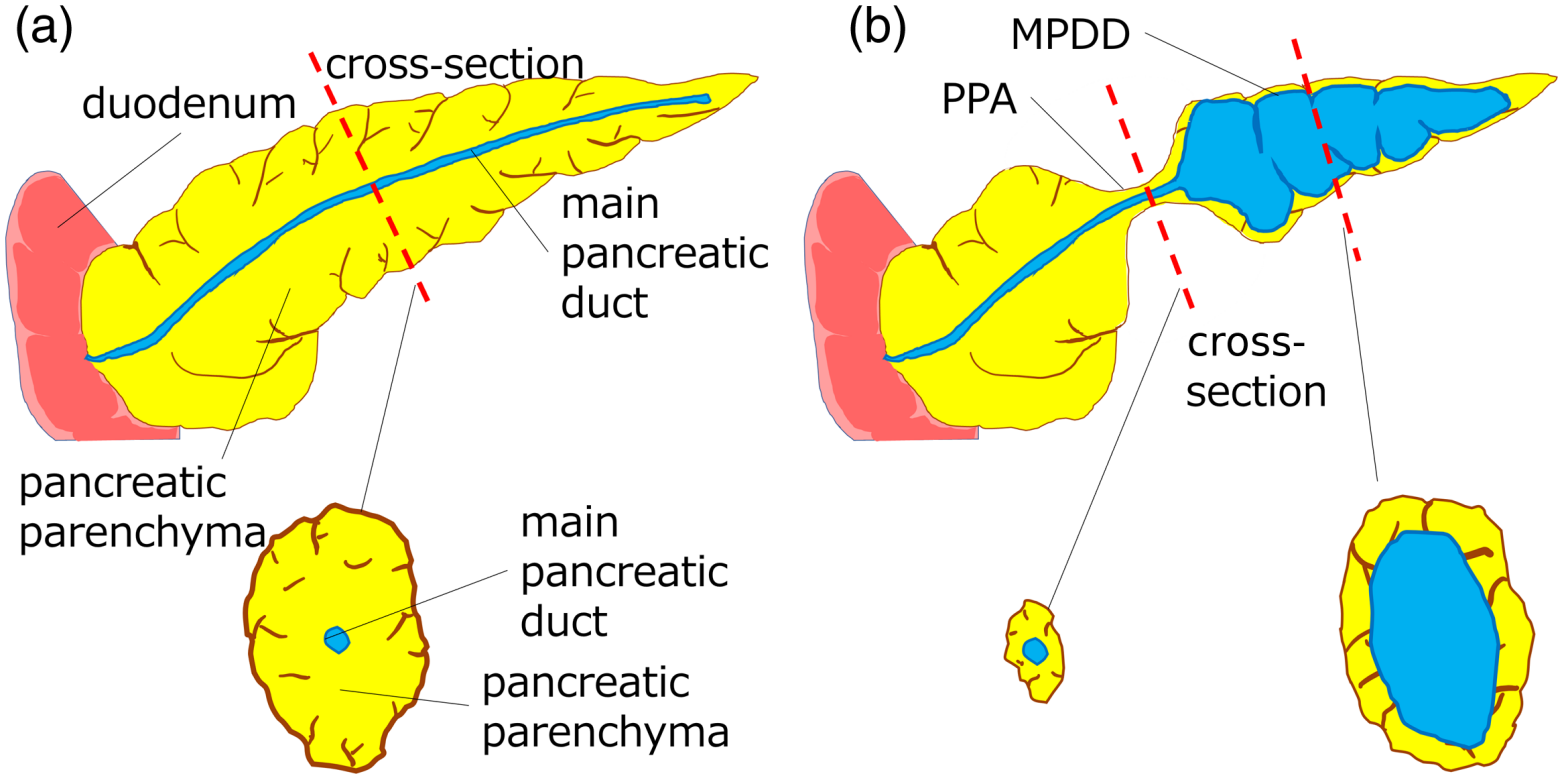
(a) Healthy pancreas and (b) pancreas with MPDD and PPA. MPDD, main pancreatic duct dilation. PPA, pancreatic parenchymal atrophy.

MPDD is an imaging finding in which the MPD is dilated by an obstruction such as a pancreatic tumor, chronic pancreatitis, or an intraductal papillary mucinous neoplasm.^21^ PPA is an imaging finding of atrophy of the pancreatic parenchyma.^22^ In this study, we focused on the cross-sectional area ratio of the pancreatic duct to the pancreatic parenchyma (DP ratio) for the detection of both MPDD and PPA. The DP ratio of cross sections in areas where the MPD was dilated or where the pancreatic parenchyma was focally atrophic. It was therefore considered that automatic calculation of the DP ratio might be useful to radiologists by helping them reduce the risk of missing MPDD or PPA (Fig. 1).

#### 2.2.2 Architecture of the proposed algorithm

**Fig. 2:**
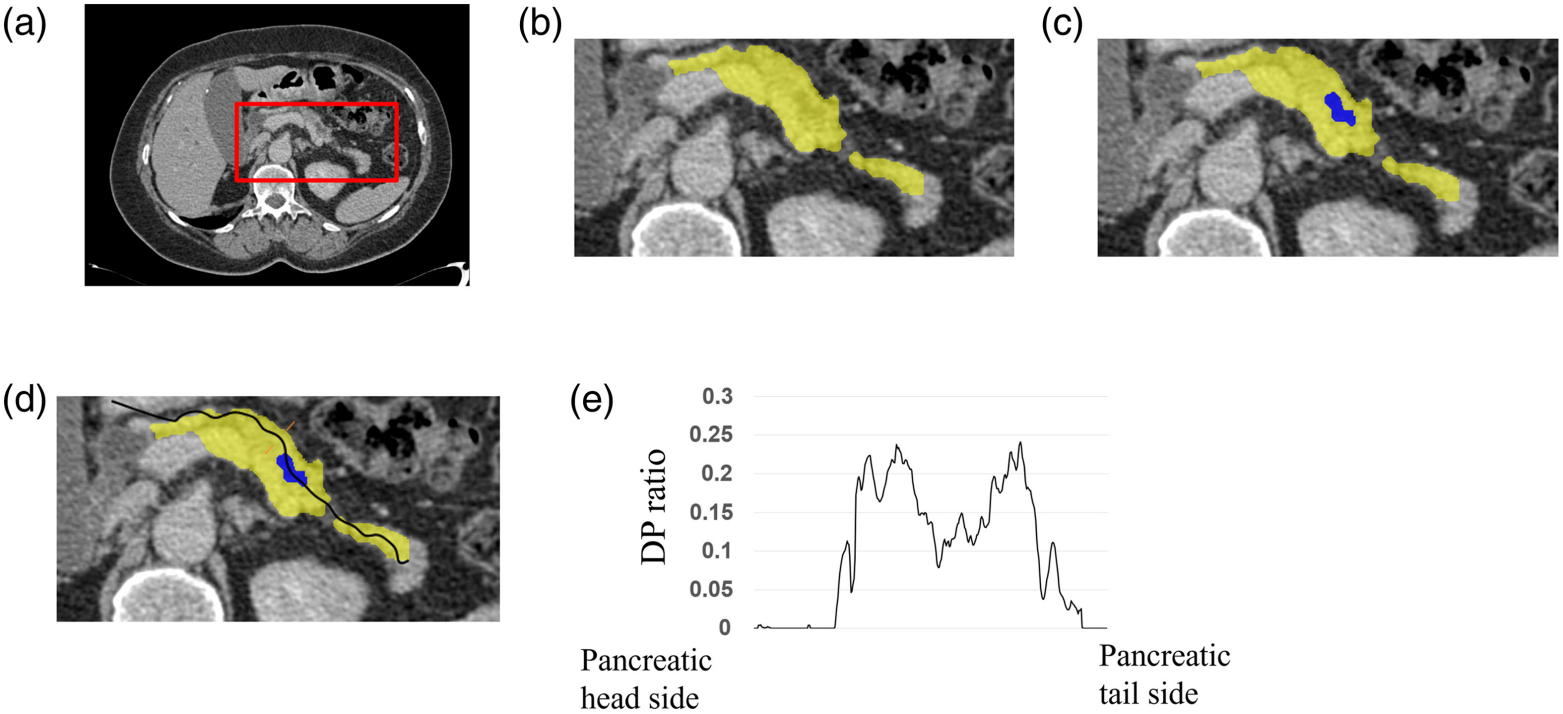
Overview of the proposed algorithm: a case with MPDD and PPA. (a) Coarse pancreas segmentation, (b) fine pancreas segmentation, (c) MPD segmentation, (d) centerline estimation, and (e) shape feature calculation. MPD, main pancreatic duct. MPDD, MPD dilation. PPA, pancreatic parenchymal atrophy. DP ratio, cross-sectional area ratio of the pancreatic duct to the pancreatic parenchyma.

Automatic calculation of the DP ratio was composed of five main processes: coarse pancreas segmentation, fine pancreas segmentation, MPD segmentation, centerline estimation, and shape feature calculation (Fig. 2).

In the process of coarse pancreas segmentation, the pancreatic region was first coarsely segmented using nnU-net and a bounding box was generated by adding a 15-mm margin in the left–right, anterior–posterior, and superior–inferior directions [Fig. 2(a)].^18^ The margin size was experimentally determined to avoid under-segmentation of the pancreatic region.

The same process from fine pancreatic segmentation to centerline estimation was performed as in Hattori et al.^23^ Fine pancreatic segmentation and MPD segmentation were performed using deep learning. Centerline estimation was performed using Dijkstra’s routing algorithm.^24^ The accuracy of each segmentation was pre-validated using different CE-CT images from the development dataset. Ground truth regions for the pancreas and MPD were created manually by engineers and confirmed by a radiologic technologist. The average of the dice scores was 0.808 for coarse pancreatic segmentation and 0.866 for fine pancreatic segmentation. The accuracy of pancreatic segmentation was improved by the two-step segmentation compared with coarse pancreatic segmentation alone. Dice scores were calculated for MPD segmentation using different 16 CE-CT images from the training data. The average of the dice scores was 0.617 for MPD segmentation.

**Fig. 3:**
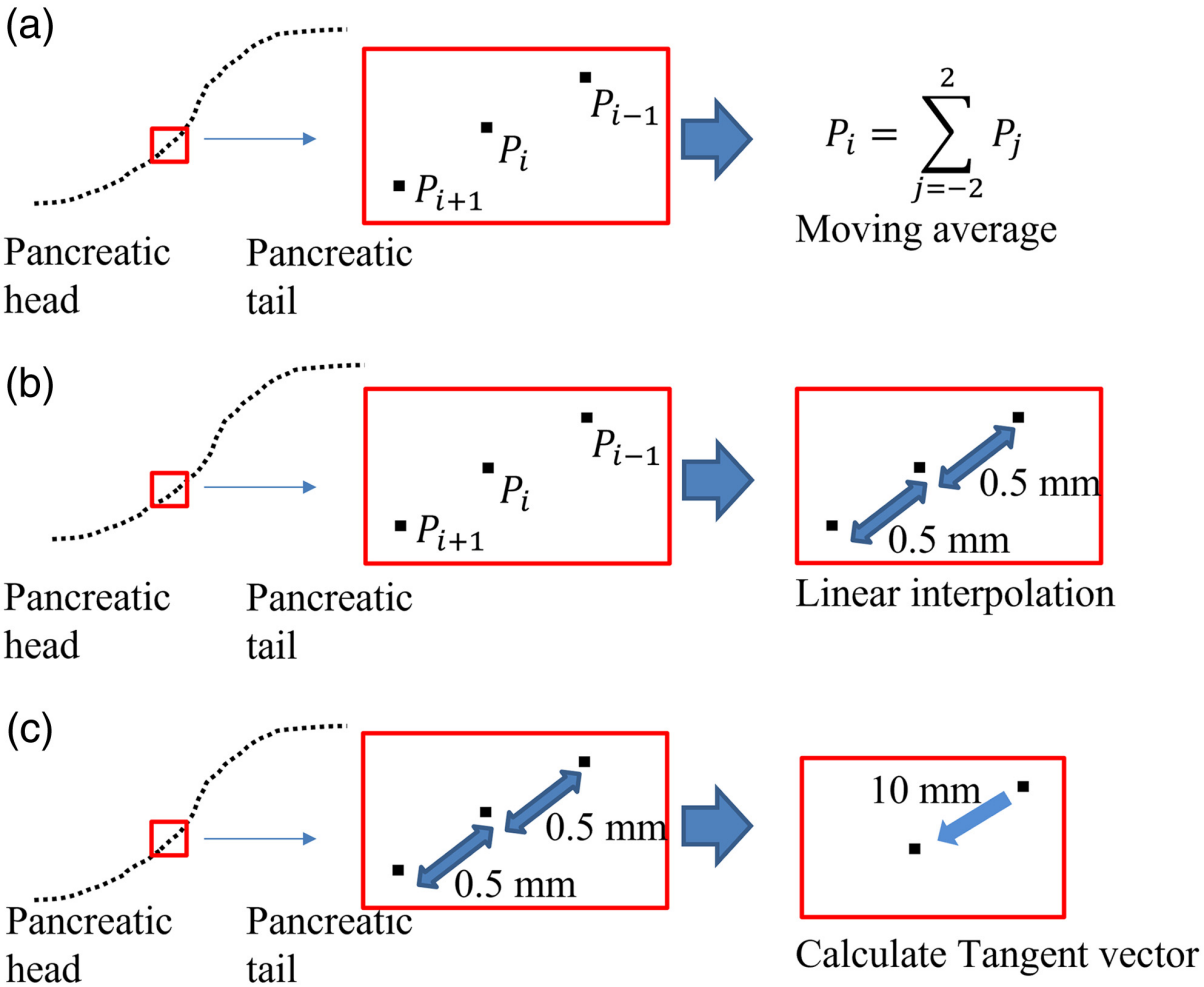
Process for the centerline: (a) smoothing the centerline by a moving average of two points before and after each point and the point itself, (b) linearly interpolating the points on the centerline so that distance between consecutive points is 0.5 mm, and (c) calculating tangent vectors from the pancreatic tail direction at 0.5-mm intervals based on the point itself and a point 10 mm ahead on the centerline.

In the process of shape feature calculation, the centerline was smoothed by a moving average of two points before and after each point and the point itself [Fig. 3(a)]. Then, the points on the centerline were linearly interpolated so that the distance between consecutive points on the centerline was equal [Fig. 3(b)]. The distance between consecutive points on the centerline was set to 0.5 mm to detect the shape changes in the pancreatic and MPD regions at the pixel spacing level of the CT images. Tangent vectors were calculated from the pancreatic tail direction at each point on the centerline based on the point itself and a point a certain distance ahead of the point on the centerline [Fig. 3(c)]. Calculating tangent vectors at 0.5-mm intervals results in a centerline that seems to be vibrating finely due to local segmentation defects. Calculating the DP ratio on a cross-section based on a finely vibrating centerline would lead to producing outliers. To prevent the influence of the vibration of the centerline, tangent vectors were calculated with reference to a point 10 mm away, which was determined empirically. The tangent vector at the last point is calculated by extending the previous tangent vector. Normal vectors and binormal vectors were identified at each point as vectors orthogonal to the tangent vectors. The cross-sections were identified based on the normal vectors and the binormal vectors at each point on the centerline. Then, the area ratio of the MPD to the pancreatic parenchyma was calculated as the DP ratio in all cross-sections [Fig. 2(e)]. When calculating the DP ratio in each cross-section, regions of the pancreas and MPD through which the centerline did not pass were removed. If the pancreas was locally not segmented or the centerline was distorted, the calculated DP ratio in those areas might be relatively high compared with other areas. Therefore, it was empirically determined to calculate the 90th percentile value of the DP ratio for all cross sections (90th DP ratio) as a representative value to detect MPDD and PPA with as little influence of incorrect segmentation and centerline as possible by observing the waveform of the DP ratio values from the pancreatic head to the pancreatic tail in a different dataset from the evaluation dataset.

The segmentation models in our proposed algorithm were trained on a workstation with 2nd Gen Intel Xeon Gold 6234 CPU, 192 GB RAM, and NVIDIA V100 PCIe GPU.

### 2.3 Evaluation

The detection performance of the 90th DP ratio for MPDD and PPA was evaluated in this study. The 90th DP ratio was calculated for the 56 abdominal CE-CT images in the evaluation dataset on a workstation with an 11th Gen Intel^®^ Core™ i7-11700 K processor @ 3.60 GHz, 32 GB of RAM, and an NVIDIA GeForce RTX 3070 GPU. Welch’s t-test was used to compare the average of 90th DP ratios calculated for the abdominal CE-CT images of 48 cases with and eight cases without MPDD and of 31 cases with and 25 cases without PPA. A p-value less than 0.05 was considered statistically significant. Receiver operating characteristic (ROC) curve analyses were also performed. Each ROC curve was obtained by modifying the threshold of the 90th DP ratio to classify cases as having each imaging finding or not. For the detection of MPDD and PPA, thresholds for sensitivity and specificity of over 90% and sensitivity and specificity at the thresholds were calculated. Moreover, Welch’s t-test was used to compare the average of 90th DP ratios of 31 cases with MPDD and PPA, 17 cases with only MPDD, and eight cases without MPDD and PPA. The Bonferroni correction was used to correct p-values for multiple comparisons in this comparison and p-value less than 0.016 was considered statistically significant.

## 3 Results

**Fig. 4:**
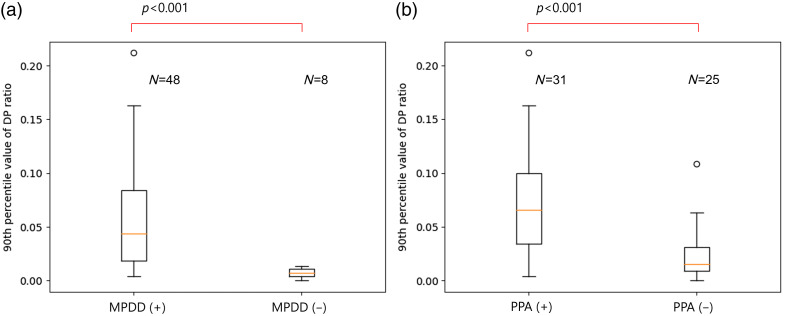
Boxplots of 90th DP ratios of (a) CT images with and without MPDD and (b) CT images with and without PPA. MPDD, main pancreatic duct dilation. PPA, pancreatic parenchymal atrophy. (+), presence. (−), absence.

**Fig. 5:**
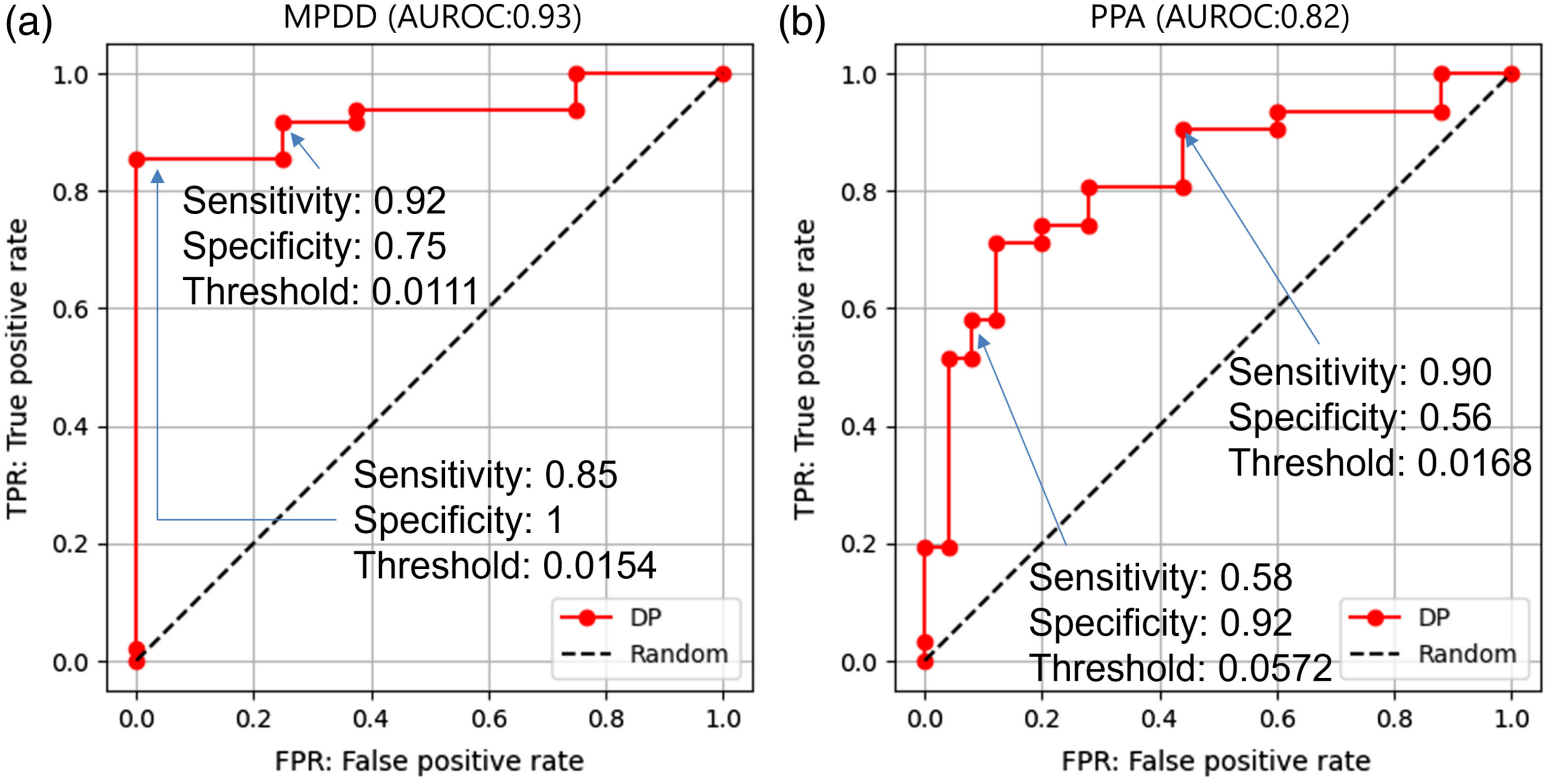
Receiver operating characteristic (ROC) curve analyses of 90th DP ratios for (a) MPDD and (b) PPA. MPDD, main pancreatic duct dilation. PPA, pancreatic parenchymal atrophy.

**Fig. 6:**
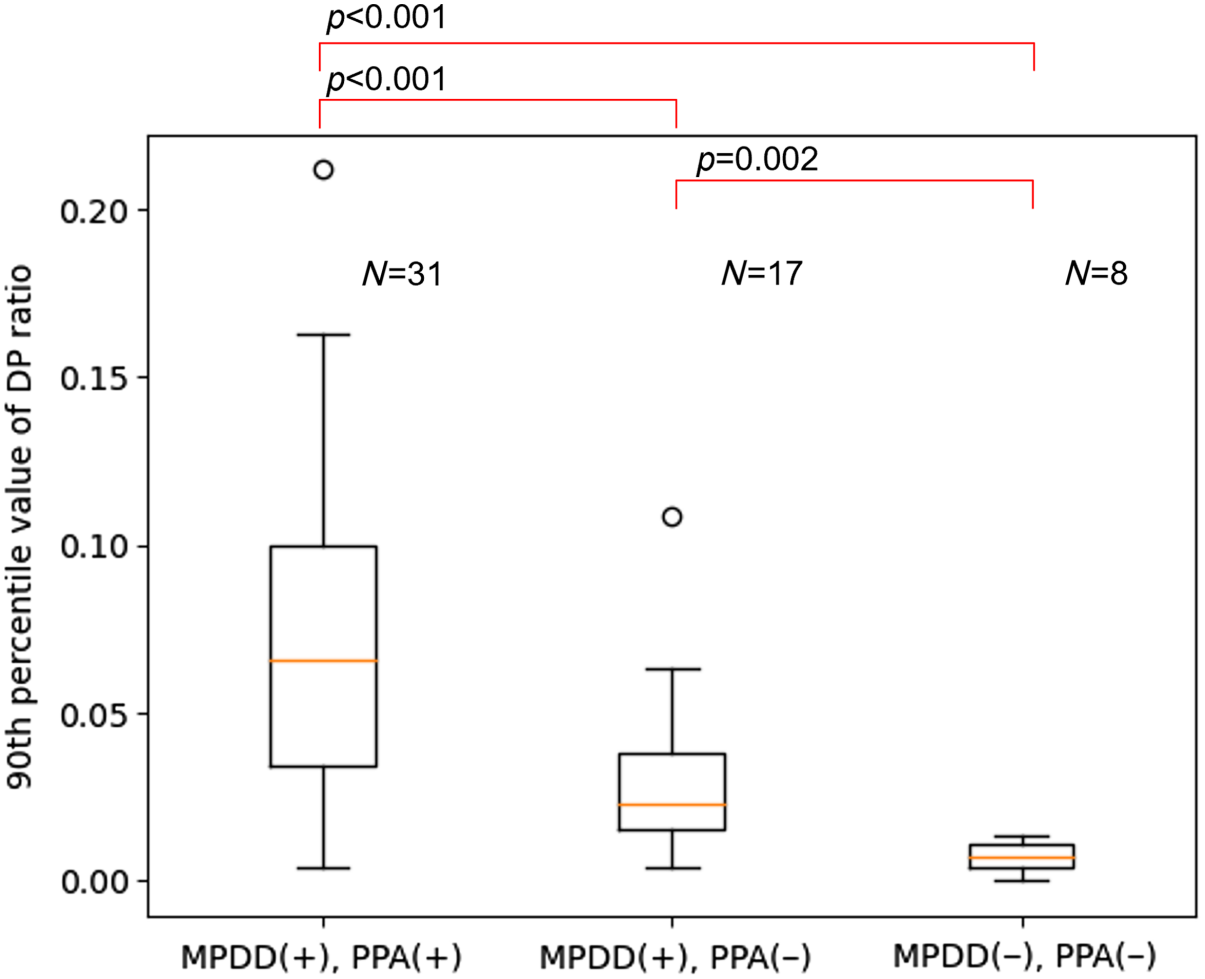
Boxplots of 90th DP ratios of CT images with MPDD and PPA, CT images with MPDD and without PPA, and CT images without MPDD and PPA. MPDD, main pancreatic duct dilation. PPA, pancreatic parenchymal atrophy. (+), presence. (−), absence.

Our proposed algorithm was applied to the 56 abdominal CE-CT images in the evaluation dataset. The results are shown in Figs. 4–6. As shown in Fig. 4, the average of the 90th DP ratio was 0.059 (0.004–0.212) in the 48 abdominal CE-CT images with MPDD, 0.007 (0–0.014) in the eight images without MPDD, 0.074 (0.004–0.212) in the 31 images with PPA, and 0.023 (0–0.108) in the 25 images without PPA. A statistically significant difference was observed in the average of 90th DP ratio between the cases with and without MPDD (p<0.001) and also between the cases with and without PPA (p<0.001). As shown in Fig. 5, the area under the ROC curve (AUROC) of the 90th DP ratio was 0.930 for MPDD and 0.823 for PPA. For MPDD detection, sensitivity and specificity were 92% and 75% with a threshold of 0.0111, respectively, and were 85% and 100% with a threshold of 0.0154, respectively. For PPA detection, sensitivity and specificity were 90% and 56% with a threshold of 0.0168, respectively, and 58% and 92% with a threshold of 0.0572, respectively. As shown in Fig. 6, the average of the 90th DP ratio was 0.074 (0.004–0.212) in the 31 images with MPDD and PPA, 0.007 (0.004–0.031) in the 17 images with MPDD and without PPA, 0.007 (0–0.014) in the eight images without MPDD and PPA. Statistically significant differences were observed in all group combinations.

## 4 Discussion

The 90th DP ratios of cases with only MPDD were higher than those of cases without MPDD and PPA, and those of cases with both MPDD and PPA were also higher than those of cases with only MPDD. Comparison of the 90th DP ratios of cases without MPDD and PPA with those of the other two groups suggests that our proposed method could detect MPDD regardless of whether PPA exists. Comparison of the 90th DP ratios of cases with MPDD and PPA with those of cases with MPDD and without PPA suggests that our proposed algorithm could detect PPA when MPDD is present. Whether our proposed method could detect PPA in the absence of MPDD was not able to be determined from our result.

There was some overlap in the distribution of 90th DP ratio values for cases with and without MPDD, and those for cases with and without PPA; hence, it can be assumed that the results of determining the presence or absence of MPDD/PPA are wrong in some cases. One cause of overlapping 90th DP ratio values is the under-segmentation of MPD. In cross-sections where MPD regions are not detected, the DP ratio is incorrectly calculated as 0, even if there is MPDD or PPA in such cross-sections. Therefore, it is incorrectly determined as having no MPDD or PPA for such cases because the 90th DP ratio is calculated as lower than it should be. Improving the MPD segmentation might lead to improving the determining the presence or absence of MPDD/PPA.

An automatically measurable shape feature is proposed for the detection of both MPDD and PPA. Nader et al. proposed a deep learning method for automatically detecting MPDD in portal venous CT images and reported that their method had an AUROC of 0.97 for detecting MPDD, which is higher performance than our method.^8^ It has been reported that there was no significant difference in the presence or absence of MPDD between benign patients and early-stage pancreatic cancer patients, but that there was a significant difference in the presence or absence of PPA.^2^ Therefore, the advantage of our proposed method for the detection of early-stage pancreatic cancer is that it could detect not only MPDD but also PPA.

The present study suffers from several limitations. First, the effectiveness of our method to detect PPA in the absence of MPDD is not clear from the present data and requires additional verification. Second, the evaluation of our proposed algorithm was based on only abdominal portal-phase CT images. Evaluation based on abdominal CE-CT images acquired in phases other than the portal phase is necessary before it can be judged suitable for clinical use.

## 5 Conclusion

We have developed an algorithm for calculating an automatically measurable shape feature called the 90th DP ratio to detect both MPDD and PPA in abdominal CE-CT images. The 90th DP ratio could be used to detect both MPDD and PPA automatically and may prove to be useful as a screening method for early-stage pancreatic cancer.

## Data Availability

The private data and code used for this article are not publicly available due to patient privacy and data-sharing agreements. The public data are publicly available by searching the articles mentioned in *References*.
